# The Identification of a Novel Spider Toxin Peptide, Lycotoxin-Pa2a, with Antibacterial and Anti-Inflammatory Activities

**DOI:** 10.3390/antibiotics12121708

**Published:** 2023-12-07

**Authors:** Min Kyoung Shin, In-Wook Hwang, Bo-Young Jang, Kyung-Bin Bu, Dong-Hee Han, Seung-Ho Lee, Jin Wook Oh, Jung Sun Yoo, Jung-Suk Sung

**Affiliations:** 1 Department of Life Science, Dongguk University-Seoul, Goyang 10326, Republic of Korea; shinmk94@dgu.ac.kr (M.K.S.); hiw910@dongguk.edu (I.-W.H.); by200015@dongguk.edu (B.-Y.J.); rudqls1211@dongguk.edu (K.-B.B.); 2021126602@dgu.ac.kr (D.-H.H.); q969@dongguk.edu (S.-H.L.); oh5929@dongguk.edu (J.W.O.); 2 Species Diversity Research Division, National Institute of Biological Resources, Incheon 22689, Republic of Korea; lycosidae@korea.kr

**Keywords:** in silico analysis, spider venom transcriptome, spider toxin peptide, antimicrobial activity, anti-inflammatory activity

## Abstract

With the increasing challenge of controlling infectious diseases due to the emergence of antibiotic-resistant strains, the importance of discovering new antimicrobial agents is rapidly increasing. Animal venoms contain a variety of functional peptides, making them a promising platform for pharmaceutical development. In this study, a novel toxin peptide with antibacterial and anti-inflammatory activities was discovered from the spider venom gland transcriptome by implementing computational approaches. Lycotoxin-Pa2a (Lytx-Pa2a) showed homology to known-spider toxin, where functional prediction indicated the potential of both antibacterial and anti-inflammatory peptides without hemolytic activity. The colony-forming assay and minimum inhibitory concentration test showed that Lytx-Pa2a exhibited comparable or stronger antibacterial activity against pathogenic strains than melittin. Following mechanistic studies revealed that Lytx-Pa2a disrupts both cytoplasmic and outer membranes of bacteria while simultaneously inducing the accumulation of reactive oxygen species. The peptide exerted no significant toxicity when treated to human primary cells, murine macrophages, and bovine red blood cells. Moreover, Lytx-Pa2a alleviated lipopolysaccharide-induced inflammation in mouse macrophages by suppressing the expression of inflammatory mediators. These findings not only suggested that Lytx-Pa2a with dual activity can be utilized as a new antimicrobial agent for infectious diseases but also demonstrated the implementation of in silico methods for discovering a novel functional peptide, which may enhance the future utilization of biological resources.

## 1. Introduction

The discovery of antibiotics has profoundly contributed to human health, reducing mortality rates by providing a means for treating infectious diseases [1]. However, the excessive dependence on and misuse of antibiotics have given rise to the emergence of multidrug-resistant (MDR) bacteria caused by spontaneous mutation or horizontal gene transfer [2,3,4]. Moreover, pharmaceutical companies have been reducing their investment in antibiotic development due to the high costs and laborious testing, which led to a decreasing number of new antibiotics entering the market [5]. Hence, it is crucial to discover novel antibiotic agents to prevent the occurrence of MDR strains and develop efficient therapeutic approaches to address infection [6,7].

Venomous species, which include spiders, scorpions, bees, snakes, and cone snails are widely spread throughout the animal kingdom. As a result of long periods of evolution, these animals produce venom with unique composition from specific tissues or venom glands [8,9]. Animal venom is composed of proteins, peptides, and ions that exert biological function and, therefore, are used for both defense and predation [10,11]. In particular, the toxin peptide of animal venom is well-known for its high molecular diversity, where sometimes a single species contains hundreds of different peptides [12,13]. The main functional properties of animal venom include neurotoxic, antimicrobial, antifungal, immunomodulatory, and wound-healing properties [14,15,16,17]. Various efforts have been made to utilize venomous components for therapeutic purposes, including Captopril originating from the *Bothrops jararaca* snake and conotoxins from cone snails [18,19,20]. Thus, animal venom can serve as a valuable platform for discovering novel therapeutic agents and molecular probes [21,22].

Peptides are a promising alternative in the pharmaceutical field, characterized by high selectivity and functional efficacy toward cellular components. They also show low cytotoxicity, low tissue accumulation, and cost-effectiveness compared with conventional medicine [23,24]. Among various naturally occurring peptides, there is substantial interest in antimicrobial peptides (AMPs) derived from animal venoms due to the fact that AMPs play an important role in the innate immune system of various organisms and protect against pathogens [25,26]. As the biological value of peptides continues to increase, the identification of novel AMPs and exploration of their functions is becoming essential for the innovative development of therapeutics.

The utilization of animal-derived resources has entered a new era with the development of in silico-based technologies [27]. Next-generation sequencing (NGS) technology has accelerated the identification of biomolecules from various biological origins while overcoming limitations of small sample sizes, such as in the case of animal venom [28,29,30]. Also, de novo assembly of an organism’s transcriptome data enables the construction of a gene library even in the absence of reference sequences, particularly when dealing with limited sample acquisition and new species [31,32]. Additionally, computational approaches such as artificial intelligence (AI)-based techniques offer analyses as well as predictions on functional, structural, and physiochemical properties [33].

The aim of this study was to identify a novel functional peptide derived from spider venom via computational approaches. This study demonstrated the discovery of the peptide with therapeutic potential that can prevent drug resistance and control infectious diseases, suggesting in silico analyses-based workflow for future discovery of drug candidates from various biological resources.

## 2. Results

### 2.1. Identification of a Novel Toxin Peptide from the Transccriptome of Spider Pardosa astrigera

**Figure 1:**
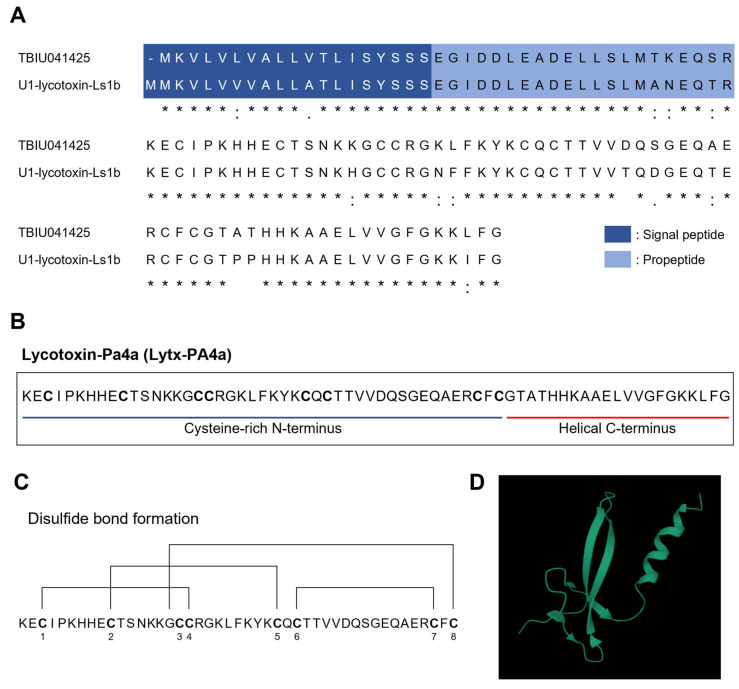
Homology analysis and structural analysis of TBIU041425. (**A**) Transcripts from the venom gland of the spider *Pardosa astrigera* were subjected to homology analysis against the known toxin peptides in the UniProt database. Among transcripts, TBIU041425 showed homology with U1-lycotoxin-Ls1b. ‘*’, ‘:’, ‘.’ indicates perfect, strong, and weak similarity between sequences, respectively. (**B**) The transcript TBIU041425 was named Lycotoxin-Pa2a (Lytx-Pa2a). The mature peptide of Lytx-Pa2a was composed of cysteine-rich N-terminus and helical C-terminus. Cysteine residues were shown as bold letters. (**C**) The predicted disulfide bonds among cysteine residues are shown. (**D**) The three-dimensional structure of Lytx-Pa2a was modeled using AlphaFold2.

In search of a novel toxin peptide, we used the venom gland transcriptome library of the spider *Pardosa astrigera* constructed in a previous study [34]. The transcripts were compared against the Uniprot database for detecting homologous sequences with the known toxin peptides. Amongst transcripts, TBIU041425 exhibited high homology with U1-lycotoxin-Ls1b from the wolf spider *Lycosa singoriensis*, showing 86.8% of identity score and 99.1% of DB coverage (Figure 1A). Both sequences possessed signal peptide and propeptide regions, which are properties of spider toxins. The mature peptide of TBIU041425 was a 66-mer peptide with two structural features (Figure 1B). The N-terminus of the sequence was rich in cysteine residues, where it was calculated that each cysteine residue forms a unique disulfide bond (C1–C4, C2–C5, C3–C8, C6–C7) discovered in toxin peptides (Figure 1C). Structural prediction revealed that each N-terminus and C-terminus of the peptide form antiparallel β-strand and α-helix, which are both structures abundantly found in toxin peptides (Figure 1D). Thus, the peptide was considered a novel spider toxin peptide and named Lycotoxin-Pa2a (Lytx-Pa2a) according to a rational nomenclature [35].

Lytx-2a was further analyzed using in silico approaches for peptide characterization. The molecular weight and net charge were calculated as 7306.46 Da and +5.9, respectively. Various toxin peptides with high net charge and helical structure often exert antimicrobial activity, whereas the distinctive secondary features often lead to other functionalities such as anti-inflammatory activity [36]. In this sense, AI-aided functional prediction was performed, resulting in positive predictions on both AMP and anti-inflammatory peptide (AIP), while having no hemolytic activity (Table 1). As the results indicated that Lytx-Pa2a is a toxin peptide with potential benefits, the peptide was synthesized for functional validation.

### 2.2. Antibacterial Activity of Lytx-Pa2a against Pathogenic Bacteria

**Figure 2:**
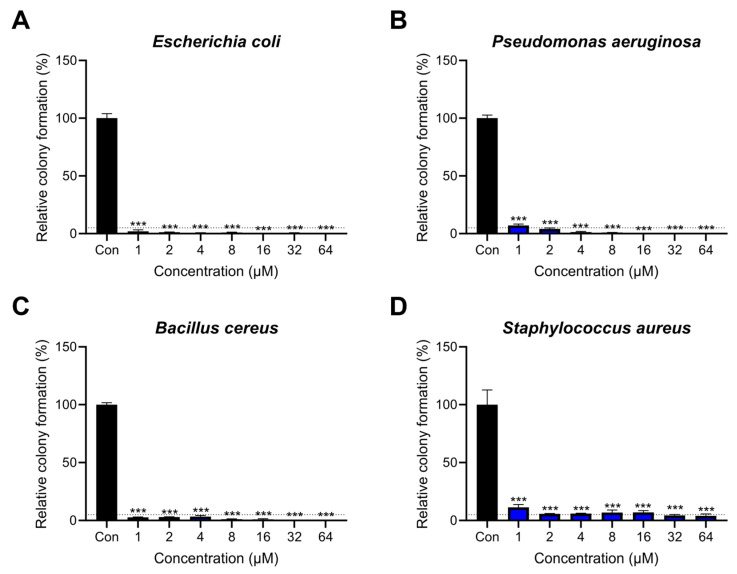
Antimicrobial activity of Lytx-Pa2a on pathogenic bacteria. Gram-negative strains (**A**) *Escherichia coli*, (**B**) *Pseudomonas aeruginosa*, and Gram-positive strains (**C**) *Bacillus cereus*, (**D**) *Staphylococcus aureus* were treated with various concentrations of Lytx-Pa2a for colony-forming assay. The dotted line is drawn at 5% of relative colony formation. *** *p* < 0.001 indicated a significant difference compared with the control.

To investigate the antimicrobial activity of Lytx-Pa2a peptides, a colony-forming assay was performed against representative pathogenic bacteria. Lytx-Pa2a peptides ranging from 1 μM to 64 μM were treated onto bacteria for 2 h to assess the colony formation. Melittin, a well-known AMP derived from bee venom, was used as a control [37,38]. The IC_95_ was determined as the concentration that inhibits 95% of the colony-forming bacterial cells. The IC_95_ of Gram-negative bacteria, *Escherichia coli* and *Pseudomonas aeruginosa,* were 1 μM and 2 μM, respectively, and those of Gram-positive bacteria, *Bacillus cereus* and *Staphylococcus aureus*, were 1 μM and 32 μM, respectively (Figure 2). Lytx-Pa2a achieved complete inhibition of growth in the higher concentrations in the case of *E*. *coli*, *P*. *aeruginosa*, and *B*. *cereus*. The IC_95_ of *S*. *aureus* was comparably high, but 1 μM of Lytx-Pa2a was enough to suppress the growth of bacteria under 10%. When compared with the results of melittin, Lytx-Pa2a exhibited similar or stronger inhibition on pathogens except for *S*. *aureus* (Supplementary Figure S1).

Moreover, the minimum inhibitory concentration (MIC) values were determined to examine the long-term effect of the peptide on pathogens. Lytx-Pa2a exhibited antimicrobial effects similar to those of melittin, with MIC values identical to those of melittin for *E*. *coli* and *B*. *cereus*. MIC of Lytx-Pa2a against *P*. *aeruginosa* was lower than that of melittin, while it was higher in the case of *S*. *aureus* (Table 2). These results demonstrated that Lytx-Pa2a showed significant antibacterial activity against both Gram-negative and -positive bacteria, and therefore, its mechanism of action was evaluated.

### 2.3. Bacterial Membrane Permeation and ROS Production by Lytx-Pa2a

**Figure 3:**
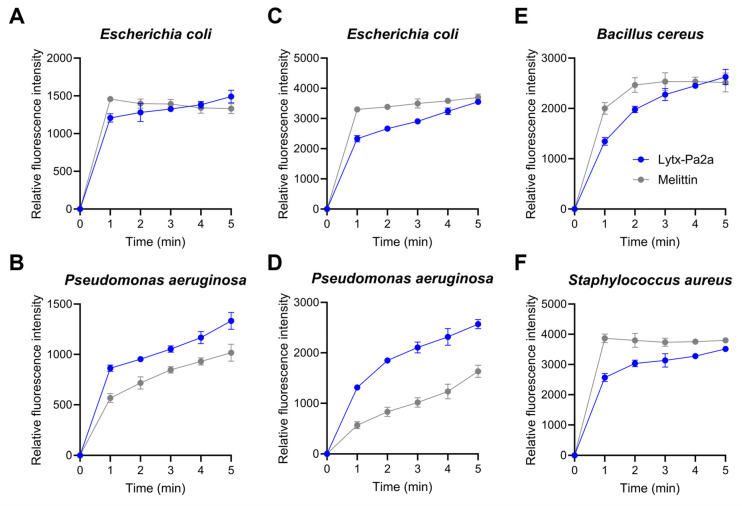
Lytx-Pa2a interacts with bacterial membranes. (**A**,**B**) N-phenyl-1-napthylamine uptake assay was performed against Gram-negative strains, *E*. *coli* and *P*. *aeruginosa*, to investigate the impact of Lytx-Pa2a on the outer membrane, which was compared with melittin. (**C**–**F**) Depolarization of bacterial cytoplasmic membrane was observed upon Lytx-Pa2a and melittin treatment using 3,3′-Dipropylthiadicarbocyanine fluorescent dye. The result of Lytx-Pa2a and melittin treatment was indicated in blue and gray, respectively.

It is well-known that AMPs interacts with bacterial membrane, leading to membrane disruption and, ultimately, cell lysis [39]. To investigate the impact of Lytx-Pa2a on the bacterial membrane, N-phenyl-1-napthylamine (NPN) and 3,3′-Dipropylthiadicarbocyanine iodide (DiSC_3_(5)) fluorescent dyes were each used to evaluated outer and cytoplasmic membrane. The fluorescence intensity was measured and compared with melittin treatment, which is known to induce bacterial membrane disruption [40,41]. When IC_95_ of Lytx-Pa2a and melittin were treated with Gram-negative strains, an immediate increase in the fluorescence signal was detected, where the signal gradually rose during the assay (Figure 3A,B). In the DiSC_3_(5)-release assay, all tested bacterial strains showed depolarization of cytoplasmic membranes upon peptide treatment, indicating DiSC_3_(5) inside bacteria cytoplasm was leaked after the Lytx-Pa2a treatment (Figure 3C–F).

**Figure 4:**
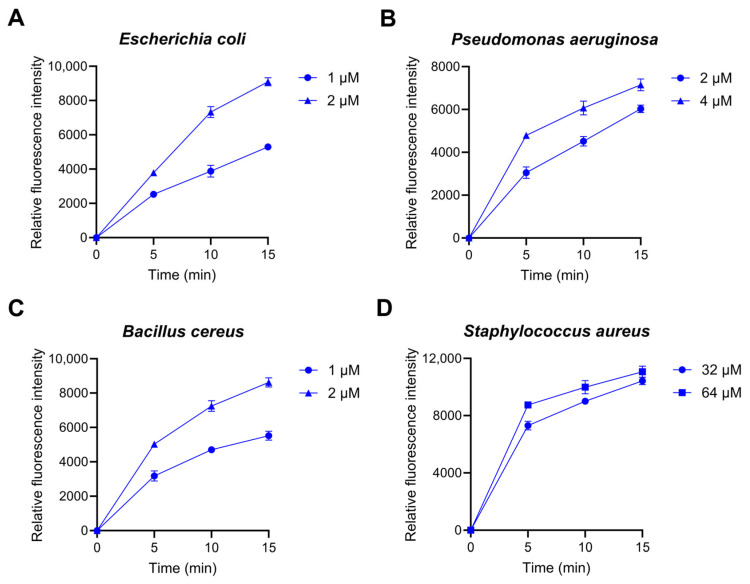
Intrabacterial reactive oxygen species (ROS) production of (**A**) *E*. *coli*, (**B**) *P*. *aeruginosa*, (**C**) *B*. *cereus*, and (**D**) *S*. *aureus* after Lytx-Pa2a treatment. ROS levels were measured once every 5 min for 15 min after treating IC_95_ and 2IC_95_ of Lytx-Pa2a.

In addition, several AMPs were reported to cause oxidative stress by interacting with bacterial components and producing reactive oxygen species (ROS). 2′,7′-dichlorodihydrofluororesin diacetate (DCFH-DA) fluorescent dye was used to measure the ROS levels in bacterial cells following the Lytx-Pa2a treatment. Relative fluorescence intensity was measured after correcting with the PBS-treated values. It was shown that ROS production was induced in all tested strains when treated with IC_95_ and 2IC_95_ of Lytx-Pa2a (Figure 4). ROS production was confirmed 5 min after starting measurement, where ROS accumulation was observed in a time- and dose-dependent manner. The overall results showed that Lytx-Pa2a disrupts bacterial membranes and promotes intrabacterial ROS production that leads to rapid and strong inhibition of bacteria.

### 2.4. Effects of Lytx-Pa2a on Mammalian Cells

**Figure 5:**
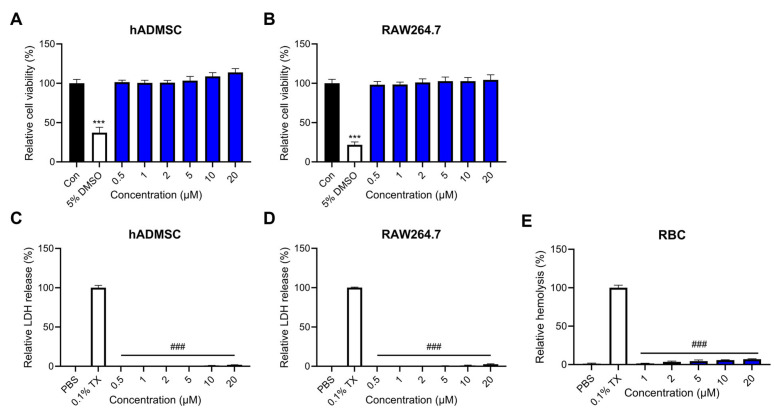
Assessment of cytotoxicity upon Lytx-Pa2a treatment on mammalian cell lines. The cell viability assay for (**A**) human adipose-derived mesenchymal stem cells (hADMSCs) and (**B**) mouse macrophage RAW264.7 was conducted to evaluate using WST-8 assay. A lactate dehydrogenase (LDH) release assay was performed to investigate the effect of Lytx-Pa2a on mammalian cell membranes. PBS and 0.1% TritonX-100 (TX)-treated group served as negative and positive control, respectively. No significant LDH release was observed in both (**C**) hADMSC and (**D**) RAW264.7 cells. Hemolysis assay showed that treatment of Lytx-Pa2a induced less than 10% of hemolysis of (**E**) bovine red blood cells compared to the positive control (0.1% TX). The result of negative control, positive control, and Lytx-Pa2a treatment was indicated in black, white, and blue, respectively. *** *p* < 0.001 compared to the control group. ### *p* < 0.001 compared to the 0.1% TX-treated group.

As cytocompatibility of peptides is crucial for therapeutic applications, we performed a cell viability assay and lactate dehydrogenase (LDH) release assay to determine the effect of Lytx-Pa2a on mammalian cell lines. Lytx-Pa2a peptides ranging from 0.5 μM to 20 μM were treated onto cells for 24 h. The cell viability assay results showed that the viability of both human adipose-derived mesenchymal stem cells (hADMSCs) and murine macrophage RAW264.7 were not affected by the treatment of Lytx-Pa2a up to 20 μM (Figure 5A,B). In addition to the cell viability test, analysis for released LDH, an indicator of cell membrane damage, was conducted (Figure 5C,D). In the case of both hADMSC and RAW264.7, no significant membrane damage was detected in every peptide-treated group, showing less than 5% compared with the positive control (0.1% Triton X-100). Lastly, to confirm the hemolytic activity, bovine red blood cells (RBCs) were treated with Lytx-Pa2a. While Triton X-100 treatment resulted in 100% hemolysis, the highest peptide concentration (20 μM) induced less than 10% hemolysis of RBCs (Figure 5E).

### 2.5. Inhibition of LPS-Induced Inflammation by Lytx-Pa2a

**Figure 6:**
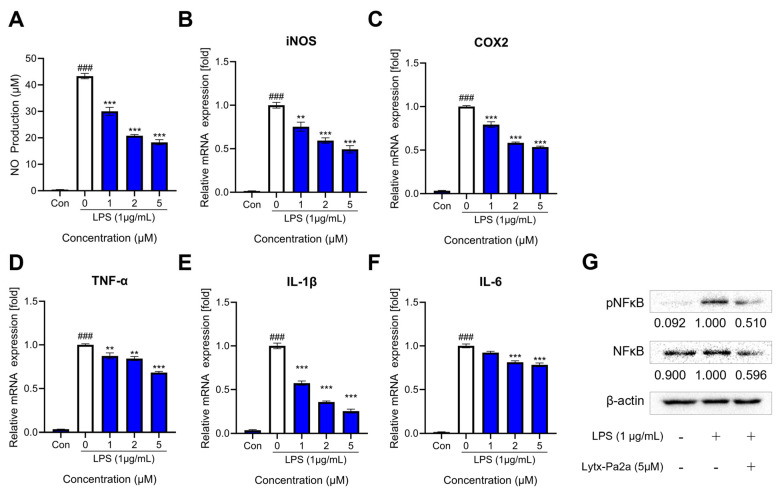
Lytx-Pa2a inhibited inflammatory mediators in LPS-stimulated macrophages. (**A**) Lytx-Pa2a treatment suppressed nitric oxide production of LPS-challenged RAW264.7 macrophages in a dose-dependent manner. (**B**–**F**) mRNA expression of inflammatory mediators, iNOS, COX2, TNF-α, IL-1β, and IL-6, were decreased by Lytx-Pa2a. (**G**) The expression and activation of NFκB were inhibited by Lytx-Pa2a treatment. The result of negative control, positive control, and Lytx-Pa2a treatment was indicated in black, white, and blue, respectively. ### *p* < 0.001 compared to the control group; ** *p* < 0.01; *** *p* < 0.001 compared to LPS-treated group.

As AI-based prediction suggested, the anti-inflammatory functionality of the peptide Lytx-Pa2a was tested using the RAW264.7 macrophage model. Macrophage were treated with 1 μg/mL LPS with or without 1, 2, and 5 μM of Lycotoxin for 24 h. It was confirmed that the peptide can inhibit the production of nitric oxide (NO), a mediator of inflammatory response (Figure 6A), up to 50% at the highest concentration. Subsequently, the mRNA expression of inflammation-related enzymes, iNOS and COX2, and pro-inflammatory cytokines, TNF-a, IL-1b, and IL-6, were evaluated using reverse transcription quantitative polymerase chain reaction (RT-qPCR). As a result, Lycotoxin-Pa2a suppressed the expression of inflammatory mediators in a dose-dependent manner (Figure 6B–F).

The nuclear factor kappa-light-chain-enhancer of activated B cells (NFκB) is a transcription factor that regulates numerous cellular pathways, including immunity, cell proliferation, and DNA transcription. When NFκB is activated by phosphorylation upon inflammatory stimuli, it can promote the transcription of pro-inflammatory mediators, including the aforementioned molecules [42]. Therefore, the protein level and activation of NFκB were investigated after a single treatment of LPS or co-treatment of Lytx-Pa2a. It was shown that Lytx-Pa2a can suppress both the phosphorylation and expression of NFκB (Figure 6G).

**Figure 7:**
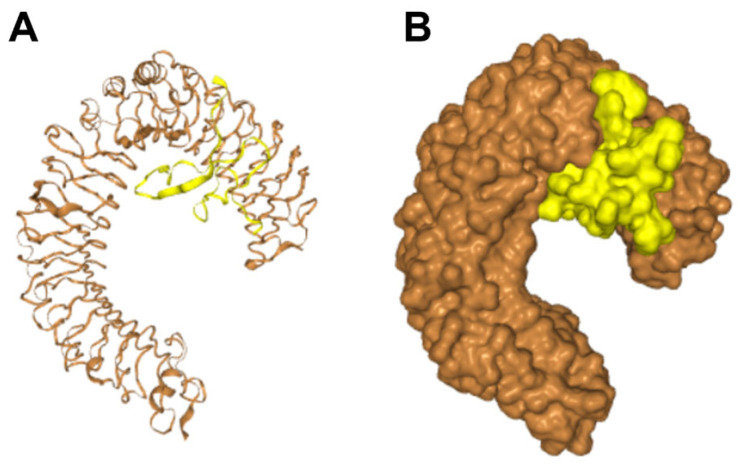
Molecular docking of Lytx-Pa2a and mouse TLR4 (PDB ID: 5IJC) was conducted using HDOCK. The generated model shows interaction between TLR4 (orange) and Lytx-Pa2a (yellow) in (**A**) cartoon and (**B**) surface version.

Toll-like receptor 4 (TLR4) is a key receptor for LPS recognition that can activate NFκB-intracellular signaling [43]. Given that Lytx-Pa2a can inhibit the NFκB pathway induced by LPS, the interaction between the peptide and TLR4 was investigated through docking simulations. Using the HDOCK server, Lytx-Pa2a was docked onto the mouse TLR4 complex (PDB ID: 5IJC), and the model with the highest binding score of −255.31 was obtained (Figure 7). Lytx-Pa2a was predicted to be located in the ligand-binding pocket of TLR4, interacting with the residues in the N-terminal involved in LPS binding [44]. These results suggested that Lytx-Pa2a exerts anti-inflammatory properties by inhibiting LPS-induced NFκB activation and has the potential to interfere with the binding of TLR4 and LPS.

## 3. Discussion

As drug-resistant microorganisms are becoming prevalent due to the misuse and overuse of antibiotics, there is a growing need for innovative and effective antimicrobial agents. Peptides present new possibilities in the pharmaceutical field as they show high diversity and selectivity and fewer side effects compared with other drugs [45]. Owing to the abundance of bioactive compounds in animal venoms, this study aimed to discover novel toxin peptides from the venom gland transcriptome of the *P*. *astrigera* spider that can potentially be developed into new antibiotics.

Animal venom is composed of peptides and proteins with a broad range of functions such as antibacterial, immunomodulatory, and neurotoxic properties [46]. Peptides are a promising candidate for next-generation drugs due to their molecular flexibility and ability to target multiple molecules [47]. Among venomous organisms, the spider is known as the taxon with the highest diversity of toxin peptides [48,49]. In particular, the venom components of spiders belonging to the *Loxosceles* and *Phoneutria* genera are associated with anti-inflammatory properties [50]. Additionally, peptides derived from spider venom with antibacterial functionality, such as the Cupiennin family, can serve as an effective means to combat antibiotic-resistant strains [51,52].

However, the relatively small quantity and difficulty in obtaining venom make the venom research difficult to achieve. Overcoming such a problem, a large amount of data can be obtained from a limited sample through in silico approaches. By utilizing the constructed *P*. *astrigera* transcriptome library, TBIU041425, a transcript with homology to a previously known toxin peptide, was secured through BLAST analysis. Signal peptide and propeptide regions were identified using a web program, followed by the identification of functional mature peptides. Structural and functional predictions provided additional biological information about the target sequence. Recent advancements in machine learning and deep learning techniques have enabled the application of various algorithms to biological analysis. Incorporating such computational methods can significantly expand the analysis and utilization of the vast biological data that has been accumulating through sequencing.

Based on structural and functional predictions, experimental verification was conducted by treating peptides to various pathogens. Lytx-Pa2a was identified as an AMP having high antibacterial functionality with low IC_95_ and MIC values. One of the major mechanisms through which AMPs exert antimicrobial activity is by damaging bacterial cell membranes [53]. Antimicrobial peptides, characterized by amphipathicity and a high net charge, can interact with the negatively charged components of bacterial cell membranes, including lipids and glycoproteins [54]. To measure changes in bacterial membrane potential following Lytx-Pa2a treatment, the fluorescent dye DiSC_3_(5) was used, where the fluorescence signal increased in a concentration-dependent manner compared with the PBS-treated control group. Additionally, when an NPN-uptake assay was performed to evaluate the effect on the outer membrane integrity of the bacteria, it was confirmed that peptide treatment led to the destruction of the outer membrane of Gram-negative bacteria. The results indicated that Lytx-Pa2a, which has a high net charge, damages both the cytoplasmic membrane and outer membrane of bacteria and induces rapid bacterial death.

Reactive oxygen species (ROS) can induce oxidative stress when interacting with bacterial components. Excessive ROS production within microbes can be fatal as it damages essential intracellular proteins and nucleic acids [55]. Using DCFH-DA to confirm ROS generation, Lytx-Pa2a was found to increase ROS levels in both Gram-positive and Gram-negative bacteria rapidly. These results show that Lytx-Pa2a can act as a multi-target AMP that induces cell death by elevating ROS along with the previously confirmed antibacterial effect through membrane permeabilization. It is possible to reduce the emergence of MDR strains through effective bacterial inhibition by the peptide, which accompanies damaging bacterial membrane integrity as well as inducing oxidative stress via targeting cellular components of bacteria.

In order to apply functional peptides for therapeutic purposes, it is essential to evaluate their effects on normal cells. Therefore, cell viability assays and LDH assays were conducted on hADMSC and mouse macrophage RAW264.7 cells to assess the cytotoxicity of Lytx-Pa2a. The results showed that treatment with Lytx-Pa2a at concentrations up to 20 μM did not significantly affect cell viability in both cell lines, indicating that the peptide had no adverse effects on normal cells within the concentration range where it can inhibit microbial growth and induce microbial death. Additionally, an LDH-release assay was performed to assess its impact on the cytoplasmic membrane of mammalian cells. LDH, a ubiquitous enzyme present in various organisms, is released when the cell membrane is damaged, providing an indicator of cell toxicity. When the Lytx-Pa2a peptide was administered, no membrane damage was detected in the tested concentration range for both cell lines. It was suggested that AMPs with a positive charge selectively interact with negatively charged bacterial cell membranes rather than neutral eukaryotic cells, indicating their ability to specifically target bacteria without off-target effects on normal cells during infection.

AMPs play important roles as host defense peptides in the innate immune system, including immunomodulatory functions [56]. Some AMPs are known to promote the production of anti-inflammatory cytokines while inhibiting pro-inflammatory cytokines, helping balance immune responses and reduce inflammation [57]. Given that Lytx-Pa2a was predicted as an AIP in previous functional predictions, an LPS-induced inflammation model was used to investigate the peptide’s anti-inflammatory potential. At the site of inflammation, COX-2 and iNOS act as major mediators that induce inflammation, and their activity leads to the formation of NO. It was observed that the production of NO and the expression of iNOS and COX-2 decreased in a dose-dependent manner upon Lytx-Pa2a treatment. Furthermore, the expression of pro-inflammatory cytokines TNF-α, IL-1β, and IL-6 also showed concentration-dependent reductions, and the activity of NFκB, a major transcription factor in the inflammatory response, was significantly suppressed. These results indicate that Lytx-Pa2a possesses characteristics of both AMP and AIP, suggesting its potential as a more effective antibiotic as it can reduce side effects caused by inflammation during pathogen infection.

Gram-negative bacteria-derived LPS is detected by the immune system, which leads to inflammatory signaling, particularly through the recognition of the lipid A by TLR4 [37]. When LPS binds to TLR4, it triggers a signaling cascade within cells, including NF-κB signaling, which activates various immune responses. This includes recruiting immune cells to the site of infection, enhancing immune responses, and facilitating the clearance of invading bacteria. However, overactivation of TLR4 can lead to excessive inflammatory responses, causing tissue damage and contributing to a chronic inflammatory state [38]. Therefore, the development of antibiotics with antimicrobial functions while appropriately regulating TLR4 activity without interfering with the overall immune response is crucial for preventing problems that can arise from excessive inflammation in infected patients. Considering that Lytx-Pa2a inhibits NFκB activity and LPS-induced inflammation, it was predicted that Lytx-Pa2a may interact with TLR4. In the following molecular docking simulation, it was predicted that the peptide is positioned on a ligand-binding pocket of the receptor. Therefore, these findings suggest that Lytx-Pa2a has the potential to alleviate symptoms and regulate infectious diseases through its anti-inflammatory functions mediated by TLR4.

In a previous study, another toxin peptide with dual antibacterial and anti-inflammatory activities, Lycotoixn-Pa4a (Lytx-Pa4a), was identified from the transcriptome data [34]. Although Lytx-Pa2a and Lytx-Pa4a showed no significant homology, both peptides are composed of cysteine-rich N-terminus with distinct disulfide patterns and helical C-terminus. In addition to inhibiting gram-positive and -negative strains, the peptides suppressed LPS-induced inflammation in macrophages. Such findings may aid understanding of the functional and sequence diversity of spider venom toxin peptides. Also, it is noteworthy that various computational approaches allowed structural analysis and functional prediction for rapid identification of a novel peptide while providing information on the anti-inflammatory activity of Lytx-Pa2a through molecular docking. As such, in silico prediction tools are useful for discovering, optimizing, and de novo designing novel functional peptides. For instance, hemolytic activity can be an indicator of toxicity and cytocompatibility of a peptide, where the selectivity of peptides is directly linked to in vivo applicability [58]. However, other physiological and structural features should be fully considered when implementing such methods, as limitations and drawbacks still exist. Despite several classifiers on hemolytic activity being available, predictions about Lytx-Pa2a were limited due to peptide length. Also, training prediction models can produce biased results depending on dataset configuration. Therefore, corroboration with rational workflow and experimental validation may aid the utilization of in silico tools in exploring novel peptides as well as biological resources.

Here, a novel toxin peptide, Lytx-Pa2a, was identified from the spider *P*. *astrigera*, and its structure and functionality were predicted using in silico analysis and AI-based tools. The peptide exhibited antimicrobial efficacy by inducing membrane disruption and ROS production, attributed to its high net charge and structural characteristics. Additionally, Lytx-Pa2a was found to possess anti-inflammatory properties by suppressing the expression and activity of pro-inflammatory cytokines and major inflammatory mediators. The results demonstrated its potential as a therapeutic agent for effectively treating infectious diseases by simultaneously inhibiting pathogens and regulating inflammation. Furthermore, Lytx-Pa2a’s rapid antimicrobial action can help prevent the emergence of MDR strains, aiding the development of next-generation antibiotics. Peptides derived from animal venom have evolved to perform specific functions, making them suitable for selective therapeutic applications. Moreover, as the synergistic effect of peptides on various diseases has been proven through combination treatment with existing drugs, peptide-based therapies can be used to complement the current drug repertoire [45,59]. In conclusion, this study offers a research approach that efficiently explores transcriptome data through computational methods, allowing for the cost-effective and rapid identification of bioactive peptides, and enhancing the efficiency of the drug discovery process.

## 4. Materials and Methods

### 4.1. In Silico Analysis of Peptide Sequence

For the identification of the spider toxin peptide, transcripts from the venom gland of *Pardosa astrigera* were first screened for homology against the known toxin peptides from the UniProt database using the Basic Local Alignment Search Tool. Signal peptides and propeptide regions were determined using SignalP 5.0 and SpiderP [60,61]. Structural modeling of the peptide and docking simulation were performed using the AlphaFold2 and HDOCK server, respectively [62,63]. For the AI-aided functional prediction, ADAM, sAMPred-GAT, AmpGram, PreAIP, PreTP-Stack, and DBAASP tools were utilized [64,65,66,67,68,69].

### 4.2. Bacterial Strains and Cell Lines

The following pathogenic strains, *E*. *coli* KCCM 11234, *P*. *aeruginosa* ATCC 9027, *B*. *cereus* KCCM 21366, and *S*. *aureus* KCCM 11335, were used in this study. Bacterial cultures were maintained in tryptic soy broth (TSB, Difco Laboratories, Detroit, MI, USA) at 37 °C under shaking condition. hADMSC and RAW264.7 were each cultured in CEFOgro™ Human MSC Growth Medium (CEFO Co., Seoul, Republic of Korea) and DMEM (Welgene Inc., Gyeongsan, Gyeongsangbuk-do, Republic of Korea) supplemented with 10% fetal bovine serum (Gibco, Grand Island, NY, USA), 1% penicillin and streptomycin (Gibco), and sodium pyruvate (Welgene Inc.). The cells were cultured in a humidified atmosphere at 37 °C with 5% CO_2_.

### 4.3. Antimicrobial Activity Assay

A colony-forming assay was performed to determine the antimicrobial activity of Lytx-Pa2a [32]. Bacterial cultures were prepared to 2 × 10^6^ CFU/mL by measuring cell numbers using optical density values obtained from a spectrophotometer and were incubated with an equal volume of peptide diluents. After 2h incubation, 100 μL of samples were spread onto a tryptic soy agar plate and then cultured overnight. The colonies were counted and presented as relative colony formation compared with the control. MIC values were determined through a two-fold microdilution method [70]. Bacterial cultures of the exponential phase were prepared into 2 × 10^6^ CFU/mL, where 50 μL of diluents were added to a 96-well plate. Peptides were serially diluted in PBS, and two-fold samples were added to each well. The plate was incubated overnight under shaking conditions, and the absorbance was measured the next day at 600 nm using a microplate reader (Molecular Devices, San Jose, CA, USA). The highest concentration resulting in no bacterial growth was considered as the MIC value.

### 4.4. Intrabacterial ROS Detection

Bacterial cultures were prepared to 1 × 10^7^ CFU/mL and were incubated with 10 μM DCFH-DA/PBS solution [71]. After 30 min, 100 μL of samples were distributed into each well of black 96-well plates. Equal volume of peptide was added, followed by the measurement of fluorescent signal at excitation of 485 nm and emission of 535 nm using an Infinite F200 Pro multimode microplate reader (Tecan, Männedorf, Switzerland).

### 4.5. Cell Viablility Assay and LDH-Release Assay

Cell viability assay was conducted to investigate the cytotoxic effect of Lytx-Pa2a on hADMSC and RAW264.7 using the Quanti-Max WST-8 Cell Viability Assay Solution (WST-8 Solution, Bio-max, Seoul, Republic of Korea). hADMSC and RAW264.7 were cultured in 96-well plates at a density of 10 × 10^4^ cells/mL. The cells were exposed to varying concentrations of the peptide for 24 h. Subsequently, the Quanti-Max WST-8 Cell Viability Assay Solution was treated into each well, and the plate was incubated for 1 h at 37 °C [72]. Additionally, the cell supernatant was used for LDH-release assay. Equal volume of samples was mixed with Dyne LDH PLUS Cytotoxicity Assay Kit (Dyne Bio, Gyeonggi-do, Republic of Korea), followed by incubation for 30 min. Dimethyl sulfoxide (Sigma-Aldrich, St. Louis, MO, USA) and 0.1% Triton X-100 (TX-100, Sigma-Aldrich) were used as a positive control for each assay. The absorbance was recorded at 450 nm using a microplate reader and the relative cell viability was determined.

### 4.6. Hemolytic Activity Measurement

To measure the hemolytic activity of the peptide, bovine RBCs obtained from Innovative Research (Novi, MI, USA) were used. RBCs were washed and diluted in PBS to prepare 4% suspension, where 100 μL aliquots were transferred to 0.6 mL microtubes. An equal volume of 2-fold peptide was added and was incubated at 37 °C for 2 h. PBS and TX-100 were used for negative and positive control, respectively. After centrifugation, supernatants from each sample were transferred to a 96-well plate, where absorbances were measured at 450 nm using a microplate reader [73].

### 4.7. NO Quatification

RAW264.7 cells were seeded onto a 96-well plate in a density of 10 × 10^4^ cells/mL [74]. After 24h, cells were treated with LPS with or without Lytx-Pa2a and cultured overnight. Cell supernatant was collected and mixed with equal volume of Griess reagent solution (Invitrogen, Carlsbad, CA, USA). After incubation in RT for 10 min, the absorbance was measured using a microplate reader at 450 nm.

### 4.8. RT-qPCR

Total mRNA was extracted from the RAW264.7 cells with TRizol Reagent (Invitrogen, MA, USA). The concentration and purity of mRNA was measured by Nanodrop-2000 (Thermo Fisher Scientific, Waltham, MA, USA). 2000 ng of RNA was synthesized into cDNA by using M-MLV Reverse transcriptase (ELPISBIO, Daejeon, Republic of Korea). The total volume of reactant was 20 μL, consisting of distilled water 7 μL, SYBR Green PCR master mix 10 μL, template cDNA 1 μL, forward primer 1 μL, and reverse primer 1 μL. The qRT-PCR were performed under the following conditions: pre-denaturation at 37 °C for 5 min, denaturation at 95 °C for 10 s, annealing at 60 °C for 30 s, and extension at 72 °C for 30 s. This cycle was repeated 40 times. The relative mRNA expression level of each gene was normalized to the control β-actin gene [75].

### 4.9. Western Blot Analysis

Total protein samples extracted from RAW264.7 cells were extracted using RIPA buffer (Biosolution, Seoul, Republic of Korea) with protease inhibitor cocktail and phosphatase inhibitor cocktail 2 and 3 (Sigma-Aldrich). The concentration of extracted protein was quantified using Pierce™ BCA Protein Assay Kit (Thermo Fisher Scientific, Waltham, MA, USA). Afterwards, 15 μL of protein was loaded and separated through 10% sodium dodecyl sulfate-polyacrylamide gel electrophoresis. Afterwards, the separated protein was transferred onto a polyvinylidene difluoride membrane. After rinsing the buffer, the membrane was blocked with 5% skim milk (Difco Laboratories) and incubated with primary antibodies. Subsequently, the membrane was washed and incubated with horseradish peroxidase-conjugated secondary antibodies. The target protein was detected using ECL Plus Western blotting detection reagents (Amersham Bioscience, Buckinghamshire, UK) and ChemiDoc™ Imaging Systems (Bio-Rad, Hercules, CA, USA), and the obtained images were quantified using Image Lab™ Software Version 4.0 (Bio-Rad) [75].

### 4.10. Statistical Analysis

All experiments were conducted in triplicate and the results are expressed as the mean ± standard error of the mean (SEM). The statistical analyses were performed using a one-way ANOVA test followed by Tukey’s post-test on GraphPad Prism 10 (GraphPad Software, La Jolla, CA, USA). *p*-values less than 0.05 were considered to indicate statisti-cally significant differences.

## Figures and Tables

**Table 1:** Functional prediction results of Lytx-Pa2a.

Antimicrobial Activity			Anti-Inflammatory Activity		Hemolytic Activity
ADAM	sAMPred-GAT	AmpGram	PreAIP	PreTP-Stack	DBAASP ^1^
0.7 (AMP)	AMP	0.9894	0.589 (AIP)	AIP	Not active

^1^ Prediction target: Human erythrocytes.

**Table 2:** MIC values for Lytx-Pa2a and melittin against pathogenic strains.

MIC (μM)	*E*. *coli*	*P*. *aeruginosa*	*B*. *cereus*	*S*. *aureus*
Lytx-Pa2a	1	1	1	8
Melittin	1	8	1	2

## Data Availability

The data of this study are available from the corresponding author (Jung-Suk Sung) upon reasonable request.

## Supplementary Materials

The following supporting information can be downloaded at: https://www.mdpi.com/article/10.3390/antibiotics12121708/s1, Figure S1: Antimicrobial activity of melittin on pathogenic bacteria. Colony forming assay was conducted on (A) *Escherichia coli*, (B) *Pseudomonas aeruginosa*, (C) *Bacillus cereus*, and (D) *Staphylococcus aureus*. The dotted line is drawn at 5% of relative colony formation. *** *p* < 0.001 indicated a significant difference compared with the control.
