# Supplementary material for: The Identification of a Novel Spider Toxin Peptide, Lycotoxin-Pa2a, with Antibacterial and Anti-Inflammatory Activities
Source: Antibiotics (Basel). 2023 Dec 7;12(12):1708. doi: 10.3390/antibiotics12121708 (PMC10740532; doi:10.3390/antibiotics12121708)
Supplement: Supplementary file 1 [file antibiotics-12-01708-s001.zip › antibiotics-2726644-supplementary.pdf]

Supplementary material

Identification of a novel spider toxin peptide, Lycotoxin-Pa2a, with antibacterial and anti-inflammatory activities

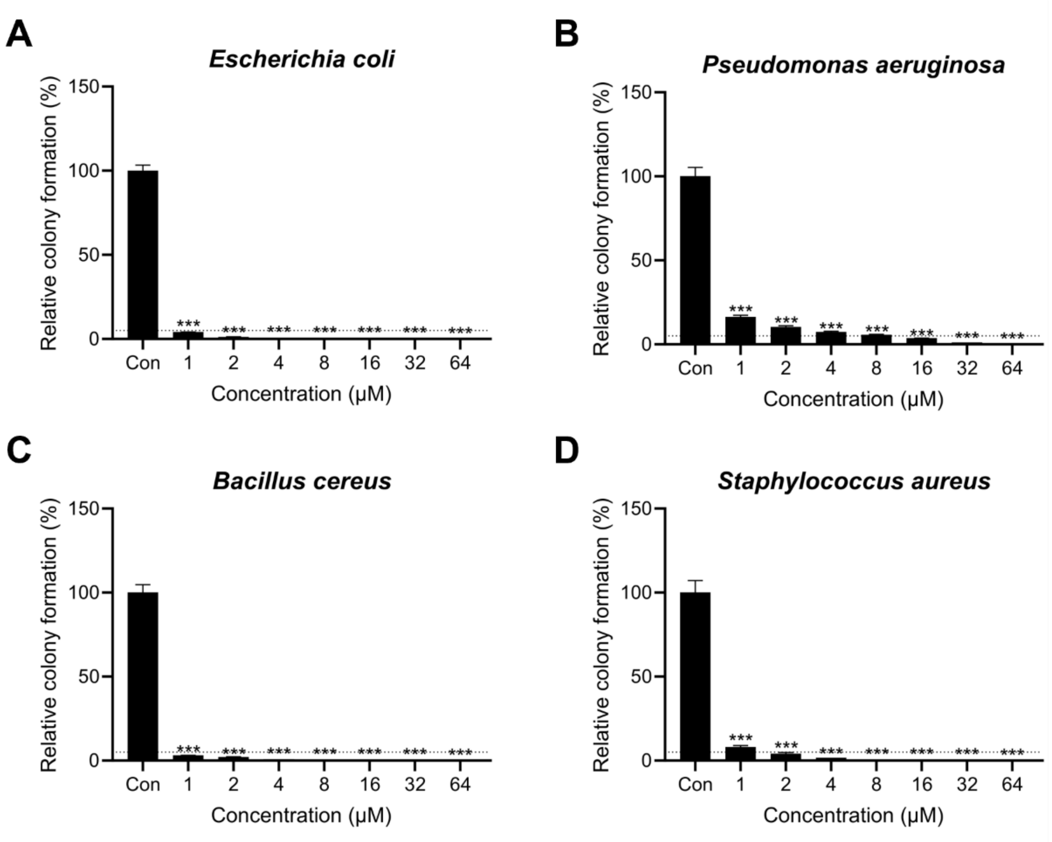

Supplementary Figure S1. Antimicrobial activity of melittin on pathogenic bacteria. Colony forming assay was conducted on **(A)** *Escherichia coli*, **(B)** *Pseudomonas aeruginosa*, **(C)** *Bacillus cereus*, and **(D)** *Staphylococcus aureus*. The dotted line is drawn at 5% of relative colony formation. *** $p < 0.001$ indicated a significant difference compared with the control.
